# Prevalence and Molecular Characterization of Cystic Echinococcosis in Livestock Population of the Malakand Division, Khyber Pakhtunkhwa, Pakistan

**DOI:** 10.3389/fvets.2021.757800

**Published:** 2021-10-21

**Authors:** Jadoon Khan, Nosheen Basharat, Salman Khan, Syed Muhammad Jamal, Sadeeq ur Rahman, Aamer Ali Shah, Sanaullah Khan, Rehman Ali, Shahid Niaz Khan, Ijaz Ali

**Affiliations:** ^1^Department of Biosciences, COMSATS University, Islamabad, Pakistan; ^2^Department of Microbiology, Quaid-i-Azam University, Islamabad, Pakistan; ^3^Department of Biotechnology, University of Malakand, Chakdara, Pakistan; ^4^College of Veterinary Science and Animal Husbandry, Abdul Wali Khan University, Mardan, Pakistan; ^5^Department of Zoology, University of Peshawar, Peshawar, Pakistan; ^6^Department of Zoology, Faculty of Biological Sciences, Kohat University of Science and Technology, Kohat, Pakistan

**Keywords:** *Echinococcus granulosus*, cystic echinococcosis, zoonosis, Khyber Pakhtunkhwa, Pakistan

## Abstract

Cystic echinococcosis (CE) is a neglected zoonotic disease prevalent in Pakistan, but the genetic diversity of the cestode is largely unexplored in the country. This study investigated the molecular epidemiology of CE infecting the livestock population of the Malakand division, Khyber Pakhtunkhwa, Pakistan. A total of 1,200 livestock, including buffaloes, cattle, goats, and sheep, were examined for echinococcosis from November 2017–2018 at different slaughterhouses in the Malakand division. Hydatid cysts were collected from different organs, and hydatid cyst fluid (HCF) was examined microscopically and used for DNA extraction. The *LSU (rrnl)* and *NAD1* genes were amplified and sequenced. The overall prevalence of CE was 17% (204/1,200), including cows (21.7%), buffaloes (17.4%), goats (10%), and sheep (9.6%). The infection was relatively more prevalent among males (17%) than females (16.9%) and animals of older age (>5 years) (*p* = 0.710). Liver (63.2%) and lungs (25%) were more affected as compared to kidneys (6.8%) and heart (4.9%). HCF analysis indicated that 52.0% of the cysts were sterile and (48.0%) were fertile. Sequencing and phylogenetic analyses confirmed 80.0% of the isolates as *Echinococcus granulosus sensu stricto* (G1-G3) in all animal species, while *Echinococcus equinus* (G4) and *Echinococcus ortleppi* (G5) were present in buffaloes. The present study concluded that CE is prevalent in the livestock population of Malakand. Besides *E. granulosus* s. s. (G1-G3), *E. ortleppi* genotype (G5) and *E. equinus* (G4) in livestock were also reported.

## Introduction

Hydatidosis or cystic echinococcosis (CE) is a zoonotic disease caused by the smallest canine cestode belonging to the genus *Echinococcus* (1). It is a neglected tropical disease (2) and is endemic in different regions of the world including the Mediterranean regions, America, Asia, Australia, Africa, and Europe (3). CE causes heavy economic losses to the livestock industry (4) including treatment cost of infected animals, decrease in milk or meat production, morbidity, and mortality (5). Economic losses in different countries have been estimated to be US$ 212.35 million in India, US$ 232.3 million in Iran, and US$ 7.708 in Turkey (6–8) and ~26.5 million Rupees in Pakistan (9).

The life cycle of the parasite involves two hosts, an intermediate host (sheep, cattle, goats, buffalo, etc.), and a definitive host (canids). Humans are considered accidental (dead-end) hosts. Among the intermediate or accidental hosts, the parasite establishes itself mostly in the liver and lungs or other soft organs and develops into a fluid-filled cyst called the hydatid cyst (4, 10).

Pakistan is an agricultural country where livestock plays an important role in annual economic growth. Agriculture contributes 18.5% to the national gross domestic product (GDP) in which the share of livestock is 11.2% (Pakistan Economic Survey, 2018–19). However, various infectious including echinococcosis and non-infectious diseases are the main impediments to the growth of the livestock sector. The prevalence of echinococcosis in different animal species ranges from 2.44 to 35% in Pakistan (5, 11–18) and shows that the prevalence increases with time. Nine different species of *Echinococcus*—*E. granulosus* s. s. (G1 to G3), *E. equinus* (G4) (19), *E. ortleppi* (G5), *E. canadensis* (G6 to G10), *E. shiquicus* (20), *E. felidis, E. oligarthrus, E. vogeli*, and *E. multilocularis* (21)—are responsible for echinococcosis in sheep, cow, buffaloes, goats, donkeys, camels, lions, cats, jackals, foxes, pigs, etc. (22). All these species have been found to infect humans except *E. equinus* (G4). The sheep strain, *E. granulosus* s. s. (G1), is responsible for most of the human cases of echinococcosis (23).

The different genotypes of *E. granulosus* responsible for echinococcosis have been reported from endemic areas throughout the world. *Echinococcus* strains reported among humans or animals from Pakistan include *E. granulosus* s. s. (G1-G3) and *E. canadensis* (G6) (5, 11, 15). Only limited studies on the characterization of *Echinococcus* species are available from Pakistan despite the immense importance of such studies in the assessment of the pathogenesis, control, and eradication of *Echinococcus* from the country. The present study investigated the prevalence and *Echinococcus* species infecting livestock in Pakistan.

## Methods

### Study Area

**Figure 1:**
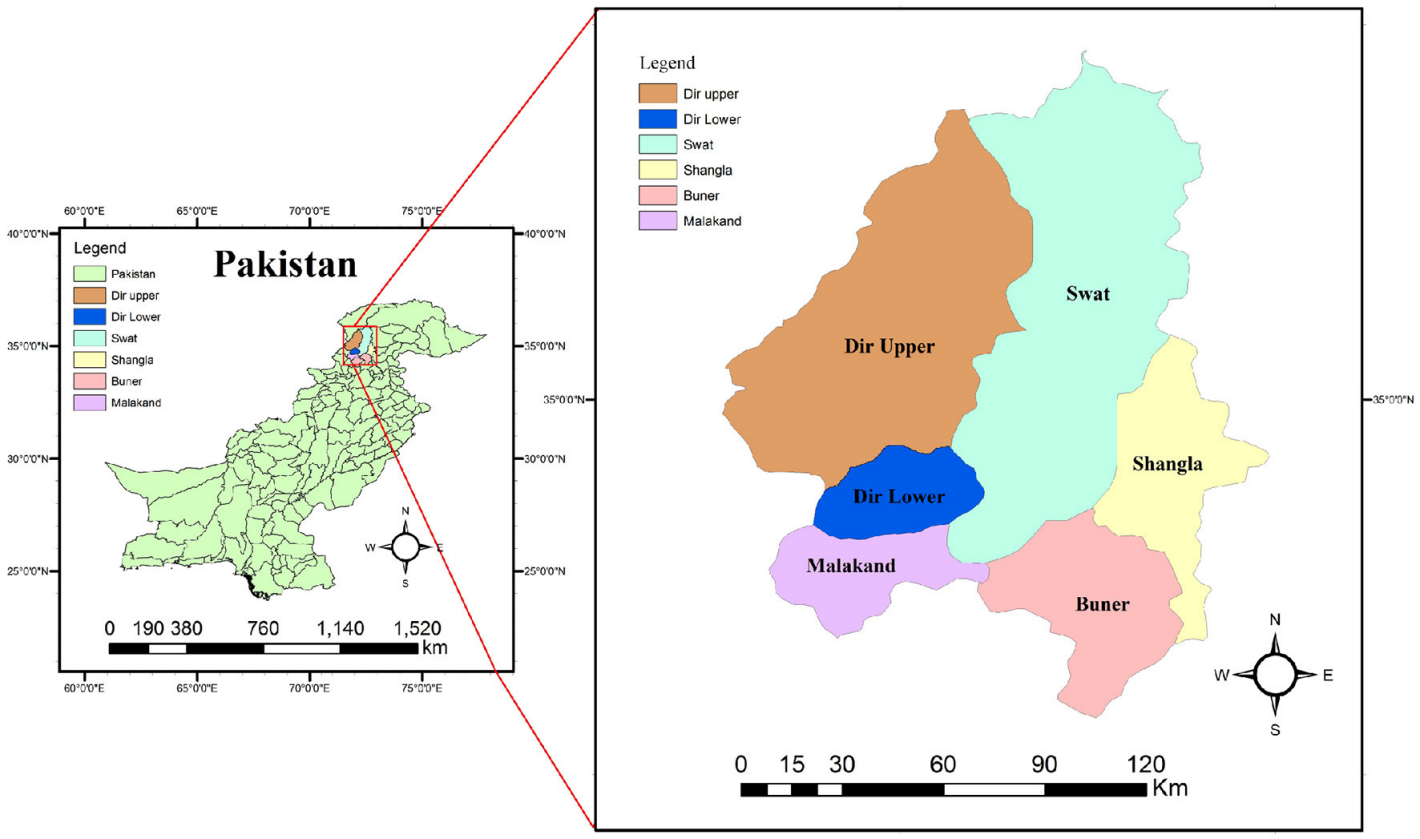
Map of Pakistan showing the study area (Malakand division).

The Malakand division is a very densely populated area, constituting 29,800 km^2^ equal to 40% area of the Khyber-Pakhtunkhwa Province of Pakistan. The study was carried out in six districts: Malakand, Buner, Swat, Shangla, Dir Upper, and Dir Lower. It borders Afghanistan in the north and northwest, while in the southwest, it shares a border with Bajaur and Mohmand Agencies of Pakistan. In the east, the Malakand division shares a border with Gilgit-Baltistan in contiguous with Xinjiang Province of China. In the south, it is attached to the densely inhabited area of Khyber Pakhtunkhwa including Charsadda, Peshawar, Mardan, and Swabi districts (Figure 1). The present study was conducted from November 2017 to 2018, and hydatid cysts were collected from livestock organs after slaughtering in the abovementioned areas of Khyber Pakhtunkhwa, Pakistan.

### Antemortem Examination

Different slaughterhouses in the selected areas of the Malakand division were visited multiple times in a month, and hydatid cysts were collected. Cysts and a detailed history of animals including animal species (cow, buffalo, sheep, or goat), cyst location in visceral organ, age, and sex were recorded. Age was asked by the middle man (buyer) and reconfirmed by examining the teeth eruption and mouthing (24). Slaughtered animals were divided into three age groups: group 1 consisted of animals of an age ranging from a few months to <3 years, group 2 included from 3 to 5 years of age, and group 3 included age above 5 years.

### Abattoir Survey and Post-mortem Examination

A total of 1,200 animals were examined, consisting of *n* = 847 buffaloes, *n* = 148 cows, *n* = 114 sheep, and *n* = 50 goats. After slaughtering, a post-mortem examination was done by visual inspection and visceral organ palpation of the carcass. Special attention was paid to the liver and lungs, although other organs were also studied/checked carefully. The animal was defined as positive if one or more cysts were found and as negative if no cyst was found (25). Hydatid cyst samples were collected under aseptic conditions from the slaughtered animals. The cyst samples were transferred in cool boxes with sterile normal saline to the Molecular Virology Laboratory, Department of Biosciences, COMSATS University Islamabad, for further experimental analysis.

### Examination of Cysts and Viability of Protoscoleces

Cyst fluid was aspirated carefully *via* a 5-ml syringe under aseptic conditions into sterile falcon tubes. The tubes were stored at −4°C until further analysis. The cystic fluid was subjected to centrifugation for 8 min at 3,000 rpm, the supernatant was discarded, and the pellet was left at the bottom. The precipitate was shacked well, and one drop was placed on a glass slide under the microscope (×40) to determine cyst fertility or sterility based on the presence or absence of protoscolices. The cyst germinal layer was also examined for broods or protoscolices under the microscope by putting a piece of layer on a slide kept in glycine. If amoeboid-like peristaltic movement (flam cell activity) was observed in microscopy, the cyst was classified as fertile. It was categorized as sterile in the absence of broods or protoscolices in examined fluid (26). Further confirmation of doubtful hydatid cysts was done by mixing of eosin dye (0.1%) with cyst fluid and staining for 15 min and then examining it microscopically if protoscolices got stained, which were considered as non-viable or, if not, were considered viable of a fertile category (27).

### Nucleic Acid Extraction and PCR Amplification

Total nucleic acid was extracted from viable hydatid cyst fluid through DNeasy Blood and Tissue Kits (Qiagen, Hilden, Germany) as per the manufacturer's instructions.

Two primers set for *LSU* and *NAD1* as *LSU*-F (5′GGTTTATTTGCCTTTTGCATCATGC3′) and *LSU*-R (5′ATCACGTCAAACCATTCAAACAAGC3′) and *NAD1*-F (5′TTATGGTAGATATTATAG 3′) and *NAD1*-R (5′ CACACACATAAAACAAGC 3′) were used for PCR amplification (11, 28). A thermal cycler (Kyratec; Model SC300G, Wembley, Australia) was used to amplify the desired genes, keeping the PCR reaction volume of about 25 μl containing PCR Master Mix (Solis Biodyne, Tartu, Estonia); 12.5 μl of MgCl_2_, dNTPs, and *Taq* DNA polymerase; forward and reverse primers (Macrogen, Seoul, Korea) of 1.5 μl each; 5.5 μl PCR water (dH_2_O); and 4 μl of extracted DNA. The following conditions were used: initial denaturation at 95°C for 5 min, followed by 95°C denaturation for 60 s, annealing at 58 and 55°C for 45 s, respectively, extension at 72°C for 45 s, and final extension at 72°C for 10 min. The reaction was completed in 35 cycles. After amplification, the PCR-amplified products were stored at −4°C for further analysis. Agarose gel (2%) was prepared, and the final PCR amplicons were separated *via* gel electrophoresis and visualized through the Gel Doc system (UVP BioDoc-It Imaging System).

### Nucleotide Sequencing and Analysis

A total of 10/96 (8.3%) PCR products of expected band size (5 for *LSU* and 5 *NAD1*) were randomly selected and commercially sequenced in both directions. For the genotypic identification, the generated sequences of each gene were aligned separately with the already known reference sequences using CrustalX 1.83 and BLAST. Maximum likelihood phylogenetic trees were constructed using MEGA-X software.

### Statistical Analysis

The epidemiological data of different variables were analyzed using the statistical tool IBM SPSS Statistics (Version 23). Chi-square Pearson's test (*x*^2^) was used for statistical analyses. The *p-*value (0.05) was considered to be statistically significant.

## Results

### Epidemiological Study

A total of 1,200 different slaughtered animals, consisting of 189 cows, 847 buffaloes, 50 goats, and 114 sheep, were studied from November 2017 to 2018. The overall prevalence of CE was recorded to be 17.0% in all animals with the highest prevalence of 21.7% in cows followed by 17.4% in buffaloes, 10.0% in goats, and 9.6% in sheep (*x*^2^ = 4.778; *p* = 0.18) (Table 1). District-wise, the prevalence was higher in Dir Lower and Malakand districts (27 and 25.5%, respectively), followed by the districts Buner (18%), Dir Upper (14.5%), Swat (10.5%), and Shangla (6.4%) (Table 2).

**Table 1:** Prevalence of cystic echinococcosis among different slaughtered animals.

**Characteristics**	**Male *n*/*N* (%)**	**Female *n*/*N* (%)**	**Total (%)**	***p*-value**	**Chi-square (*x*^**2**^)**
Cow	22/92 (24.0)	19/56 (34.0)	41/189 (27.7)	0.18	4.778
Buffalo	53/222 (24.0)	94/478 (19.6)	147/847 (21.0)		
Goat	2/15 (13.3)	3/30 (10.0)	5/50 (11.1)		
Sheep	3/44(7.0)	8/59 (13.5)	11/114 (10.7)		

*n, infected animals; N, total observed*.

**Table 2:** Prevalence of cystic echinococcosis among different districts of the Malakand division.

**District**	**Animal observed**		**Total**	**Prevalence (%)**
	**Positive**	**Negative**		
Swat	21	179	200	10.5
Buner	36	164	200	18.0
Shangla	13	187	200	6.5
Malakand	51	149	200	25.5
Dir Upper	29	171	200	14.5
Dir Lower	54	146	200	27.0
Total	204	996	1,200	17.0

The sex-wise distribution of CE showed no difference in prevalence between male (17.0%) and female (16.9%) animals. Similarly, no difference in prevalence was observed among animals in the various age groups (*x*^2^ = 0.321; *p* = 0.85) (Table 3).

**Table 3:** Prevalence of cystic echinococcosis in different age groups of organs of livestock.

**Characteristics**	**Cows *n*/*N* (%)**	**Buffaloes *n*/*N* (%)**	**Goats *n*/*N* (%)**	**Sheep *n*/*N* (%)**	***p*-value**	**Chi-square (*x*^**2**^)**
**Age-wise prevalence**						
Age group 1 (below 3 years)	8/19 (42.1)	36/167 (21.5)	-/-	-/30	0.85	0.321
Age group 2 (3–5 years)	18/108 (16.6)	58/240 (24.1)	2/21 (9.5)	3/45 (6.6)		
Age group 3 (above 5 years)	15/21 (71.4)	53/293 (18.0)	3/24 (12.5)	8/28 (28.5)		
**Organ-wise prevalence**						
Liver	13/148 (9.0)	115/700 (16.4)	-/45	1/103 (1.0)	0.001	0.994
Lungs	23/148 (15.5)	23/700 (3.3)	3/45 (6.6)	2/103 (2.0)		
Kidney	4/148 (2.7)	6/700 (1.0)	1/45 (2.2)	3/103 (3.0)		
Heart	1/148 (0.7)	3/700 (0.4)	1/45 (2.2)	5/103 (5.0)		

*n, infected animals; N, total observed*.

According to the organ susceptibility to hydatid cysts, the liver was found to be more infected (63.2%) than the lungs (25.0%), kidneys (6.8%), and heart (4.9%). The distribution of CE among the various organs varied significantly (*x*^2^ = 0.994; *p* = 0.001) (Table 3). Microscopic observation of the hydatid cyst fluid (HCF) showed that 106/204 (52%) were sterile, while 98 (48%) were fertile. Histopathological observation of hydatid cyst in the liver showed a thickening of the hepatocyte tissue and proliferation of the fibrous wall around the cyst as well as thickening of the bronchiolar and alveolar wall in the lungs.

### Echinococcus Species Identification

**Figure 2:**
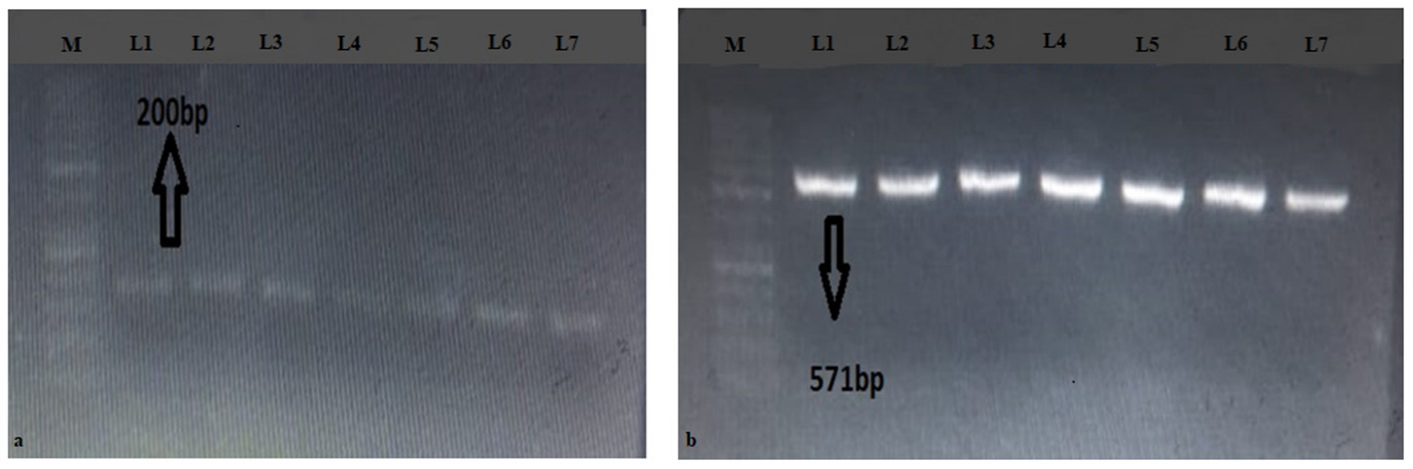
Gels of **(a)** *LSU* (rrnl) and **(b)** *NAD1* genes. M represents 50 bp DNA ladder; L1-L7 represent positive samples.

Upon PCR amplification, 96/98 amplicons produced bands on both *NAD1* and *LSU* (rrnl) of 200 and 571 bp, respectively (Figure 2). In total, 1 sample out of 98 (1.02%) was amplified for *NAD1* and 1 (1.02%) for *LSU* (rrnl).

### Sequence Analysis

**Figure 3:**
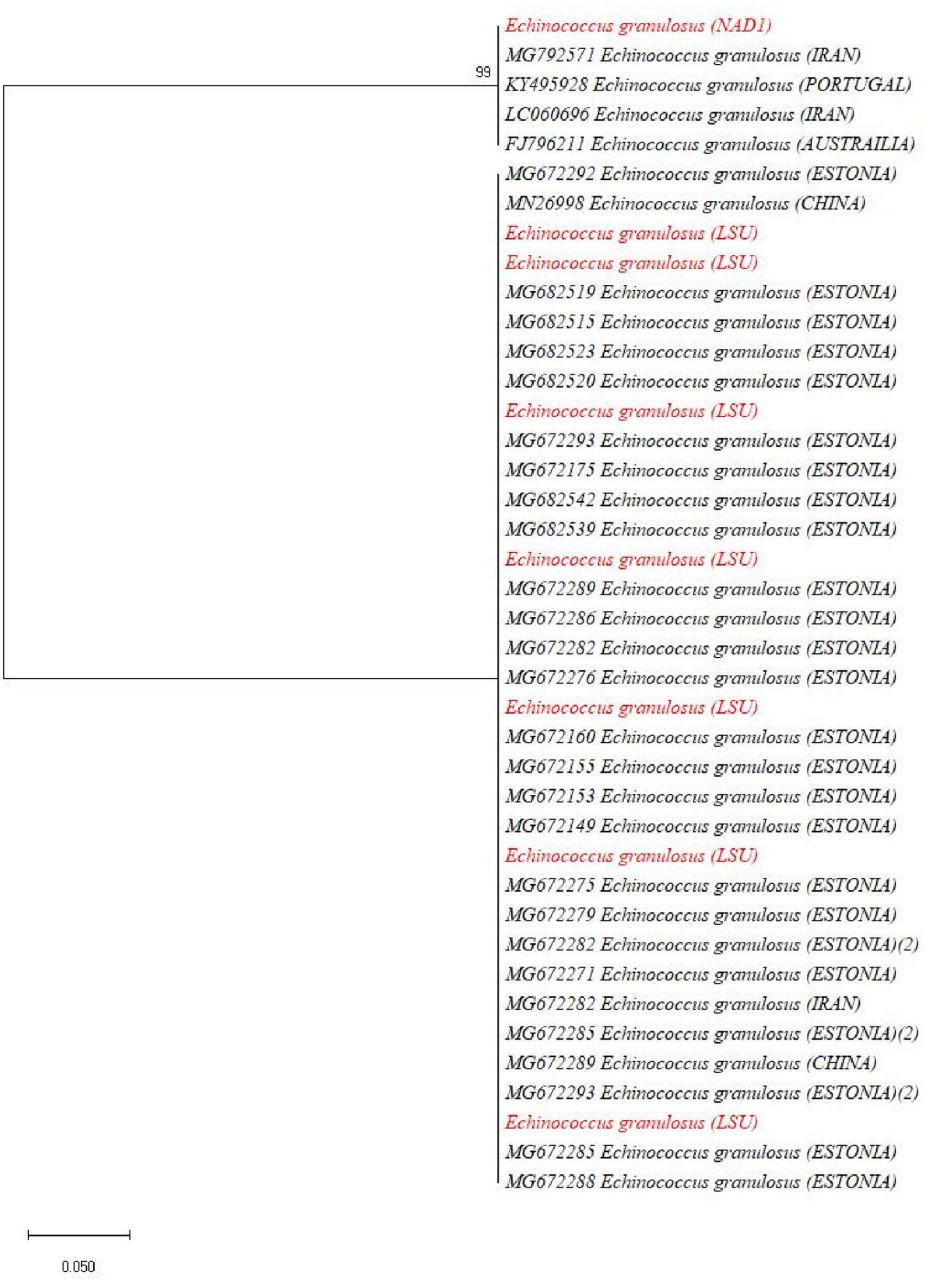
Maximum likelihood phylogenetic tree showing *Echinococcus granulosus* (G1-G3) genotypes from the livestock population of the Malakand Division, Khyber Pakhtunkhwa, Pakistan, based on nucleotide sequences of the *LSU* (rrnl) and *NAD1* genes.

**Figure 4:**
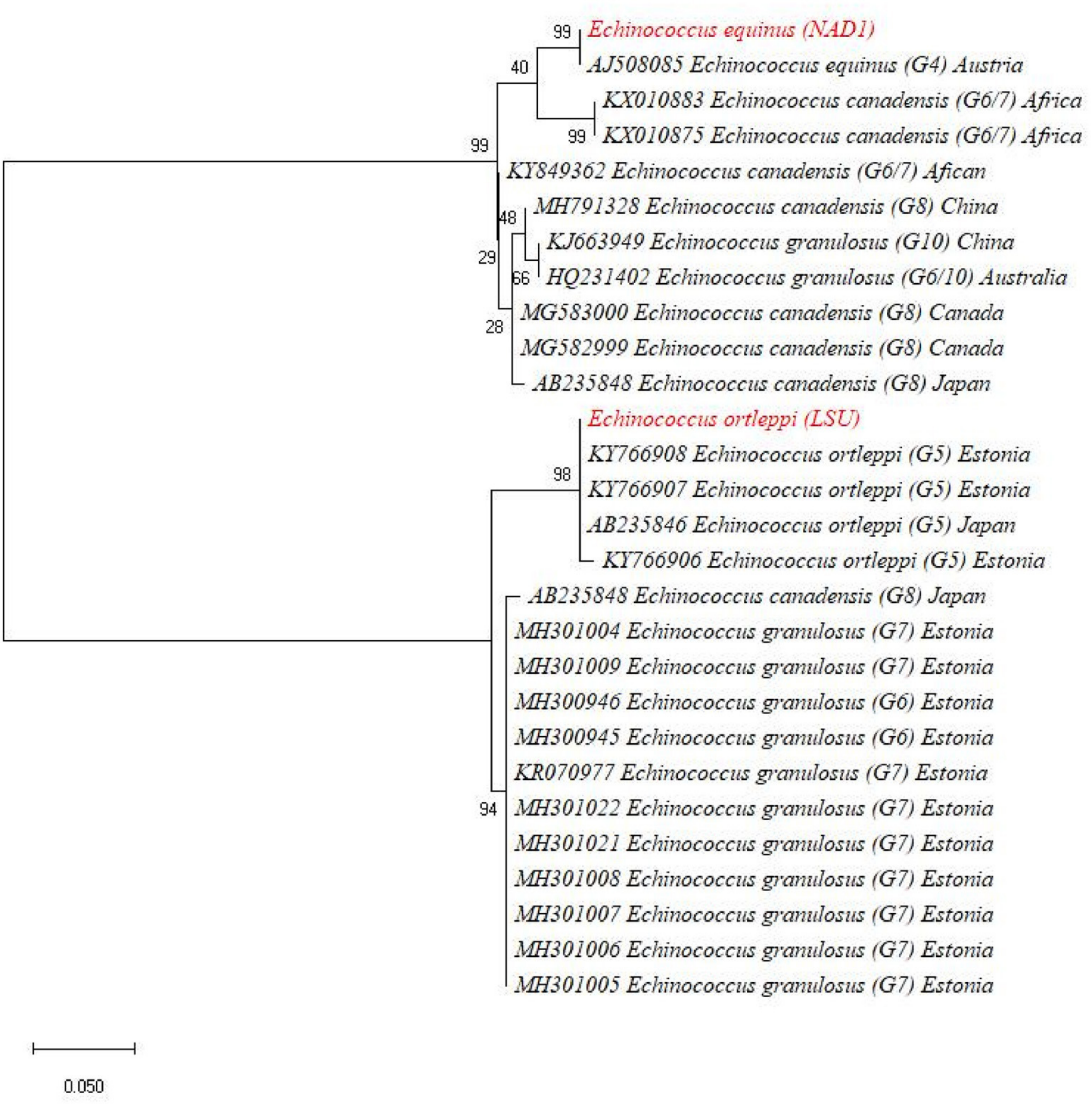
Maximum likelihood phylogenetic tree showing *Echinococcus ortleppi* (G-5) and *Echinococcus equinus* (G4) from Khyber Pakhtunkhwa, Pakistan, based on nucleotide sequences of the *LSU* (rrnl) and *NAD1* genes.

The BLAST results identified the eight isolates [based on both *NAD1* and *LSU* (rrnl) sequences] as *E. granulosus* s. s. (G1-G3) in four different animal species including cows, buffaloes, goats, and sheep. The one-cyst sample which only gave amplification of *LSU* (rrnl) from buffalo showed 99.33% sequence identity to *E. ortleppi* (G-5) of the previous deposit (accession number: KY766908) while the other one cyst which gave amplification for the *NAD1* gene only from buffalo showed 99.33% sequence identity to *E. equinus* (G4) (accession number: AJ508085). In order to ascertain the relationship of our isolates with other globally reported *E. granulosus* genotypes, a phylogenetic tree was constructed and revealed that our eight samples [four for *NAD1* and four for *LSU* (rrnl)] clustered with *E.granulosus* s. s. (G1-G3) (Figure 3). In the phylogenetic tree, the one *NAD1* sequence clustered with *E. ortleppi* (G5) and the *LSU* (rrnl) sequence clustered with *E. equinus* (G4) Austria (Figure 4). The sequences obtained in the current study were submitted to NCBI GenBank with the BioSample accession SAMN20286669.

## Discussion

Cystic echinococcosis is a parasitic infection with a worldwide distribution, producing substantial health and monetary losses (29). CE has been merely reported in Pakistani slaughtered animals, and only a few reports are available (9, 12, 14, 15, 17, 25, 30–33). The reported prevalence in slaughtered animals from India, Italy, Ethiopia, China, and Iran was 0.58 (34), 10.6 (35), 31.4 (36), 11.84 (37), and 10.7% (38), respectively. CE prevalence varies from region to region and in different provinces of Pakistan. The reported prevalence is quietly high in different regions of Punjab such as 31.05 (39), 33.0, (30, 31), 60.6 (40), 35.0 (12), and 6.67% (15). However, in Khyber Pakhtunkhwa province the reported prevalence was 14.57 (9) and 9.0% (25) in slaughtered animals. The prevalence of 17.0% was recorded in the current study in livestock population of the Malakand division. The variation in the presence of a disease may be due to the political attachment, which means that the disease requires either some public funds, publicity, management, or involvement of other government agencies (41). In opposition to the political intervention, the epidemiological characterization of the disease, such as endemic (disease either stable), epidemic (increasing), or exotic, is also one of the necessary parameters. Furthermore, those diseases which are considered as production diseases (affect the profitability as well as productivity) are thought to be the livestock owner matter individually or collectively (41). Some additional factors like culture variation, social activities, geographical distribution, and variation in approach toward hygienic conditions, husbandry practices, and relation with dogs (42) may also greatly influence the prevalence rate.

In the case of CE infection, intermediate hosts are herbivores like cows, buffaloes, goats, sheep, and camels (29). Getaw et al. (36) from Ethiopia recorded that CE was more prevalent in cows 46.8% followed by 29.3% in sheep and 6.7% in goats. Vaidya et al. (43) and Pednekar et al. (34) showed similar findings. A study by Li et al. (37) found a 15.59% prevalence in sheep followed by a 9.15% CE positivity among cows. These findings were in agreement with Qinling et al. (44). Likewise, Tabar et al. (45) found that the prevalence of CE among buffalos was significantly higher than that of other species. Our study confirmed the highest prevalence of CE in cows (21.7%) followed by buffaloes (17.4%), goats (10.0%), and sheep (9.6%). A report from the adjacent area showed the highest prevalence in buffaloes (15.88%), cows (15.79%), sheep (15.38%), and goats (3.25%) (9). From the livestock of Punjab, the highest prevalence rate of CE (35.0%) in cows was reported by Anwar et al. (12), while Latif et al. (15) reported the highest prevalence rate of hydatid cysts (7.52%) in sheep and (7.19%) in buffalo followed by goats and cattle (5.18%). Mustafa et al. (17) from Punjab and Ahmed et al. (33) from Baluchistan illustrated the same observations. The different studies coincide with our study of a higher prevalence rate of echinococcosis in cows and buffaloes, which may be due to the variation in the sale and purchase as well as slaughtering behavior of higher number as compared to other animals. The lowest prevalence in goats and sheep may be due to the grazing behaviors as they mostly ingest the upper part of shrubs, i.e., less contaminated with eggs of echinococcosis which may decrease the chance of infection (9).

Echinococcosis can infect both sexes and is found in every age group (9). In the present study, the observed infection rate in females and males was 16.9 and 17.0%, respectively. Similar results were observed in different reports from Pakistan, showing that females were more infected than males (9, 12, 14). These findings were also in agreement with Banda et al. (46) and Lemma et al. (47). It was concluded from all the reports that the higher prevalence rate of CE in females may be because of the higher slaughtering of older females than males. Generally, the females are maintained for a longer time to give offspring several times. Oppositely, the males are mostly slaughtered at young ages not more than 2 years; therefore, the CE may not be developed to diagnose because of its small size (48). Besides the long duration of exposure to infection, most of the animals are cast off when the yield of their milk is reduced which may be due to a long time of exposure to echinococcosis. Age-wise distribution by Li et al. (37) revealed that the increase in age significantly leads to an increase in the prevalence of hydatid disease; analogous to this report, Haleem et al. (9) also showed that animals with mostly <1 year have less infection rate (11.29%) followed by 28.26% in older animals of age more than 5 years. However, the current study showed that a 16.9% rate of prevalence in the livestock population belongs to those of age below 3 years and 16.4% to those having age between 3 and 5 years while 17.8% to those having age above 5 years. The difference in the prevalence of echinococcosis among different age groups may be due to a higher risk of infection and reinfection due to low immunity as well as the availability of sufficient time required for the development of mature cyst which may need 4–13 months to grow in diameter of a few millimeters (49). In this way, the diagnostic techniques may fail to detect all the cysts in an organ, that is why the early cyst may escape from detection during inspection time. Henceforth, the sensitivity of the diagnostic methods increases with the increase in the age of the animals (48).

The larval form of echinococcosis, the hydatid cyst, can be found in various organs like the liver, lungs, kidney, heart, spleen, bones, and brains. As shown from the findings of the current study, the liver is the most affected organ (63.2%) followed by the lungs (25%), kidneys (6.8%), and heart (4.9%). A recent study from the adjacent area of the same province reported similar supporting results of higher prevalence (63.49%) of cysts in the liver, 23.80% in lungs, and 2.64% in mesentery while 10.05% of the cyst involved the heart and kidney (9). Similar supporting results of higher infection in the liver followed by the lungs, kidney, and heart are also published globally by Qingling et al. (44) and Tabar et al. (45). In distinction to our results, the highest percentage of the cyst was 47.31% in the lungs, 25.31% in the liver, and 1.83% in the spleen while a single cyst from the kidney and heart was also observed (12). Similar findings were found in Mustafa et al. (17) and Khan et al. (14); however, Getaw et al. (36) and Ahmadi and Meshkehkar (50) reported different findings. According to various studies in support of our results, the highest infection in the livers may be because the liver receives digested material firstly through a hepatic portal vein which may contain the oncosphere which shows high tropism for the liver (51), and mostly it traps there making a cyst; however, during some circumstances, it escapes and targets other organs like the lungs, hearts, kidney, and brain (52).

Some of the cysts show viable protoscolices and hence are known as fertile, while others do not and are hence called sterile (22). Different studies report a different percentage of fertile and sterile cysts, like a study from India which reported 59.04% of fertile cyst followed by 40.95% of sterile cysts (34). Studies done on the livestock population of Punjab show that fertile cysts are higher in number compared to sterile cysts (12, 15, 17, 33), while Haleem et al. (9) show that sterile cyst numbers are higher than fertile cysts followed by calcified cysts as 53.43, 29.10, and 17.46%, respectively. Our findings show that 52% were sterile while 48% were fertile, which may be due to the variation in strains of Echinococcus, variation in the feeding behavior of the animals, preference of their host, pathogenesis, infectivity, and rate of development of the cyst, and that sterile cysts may be due to degenerative atrophy caused by the reaction of the body against various cysts, which may lead to caseation and calcification followed by degeneration (38, 53). Hydatid cysts present in a different geographical area, host variability, size, and site have different fertility rates (54).

A complex of different genotypes of the *E. granulosus sensu lato* differs in the development, sensitivity, pathology of infection to anthelminthics, and intermediate specificity of hosts including camel, cattle, sheep, horse, pigs, and dogs. Different mitochondrial gene studies identified genotypes G1-G10 by using NADH dehydrogenase (nad) and cytochrome C oxidase (cox) genes (55). *E. granulosus* is cosmopolitan in geographical distribution and is common in domestic animals in the South and Central regions of America, Africa, Asia, and Mediterranean regions including Lebanon, Iran, Kuwait, Iraq, Syria, Saudi Arabia, Jordan, and Pakistan (56, 57). Globally, G1-G3 are predominant genotypes infecting cattle. Few studies reported G5 from cattle in France, Argentina, Italy, Brazil, South Africa, and Sudan. The G6 genotype was found in Libya, Sudan, and Egypt (58). Pakistan has four genotypes of *Echinococcus*, including *E. granulosus s. s*. (G1-G3) and *E. canadensis* (G6), as reported by Ali et al. (11), Ehsan et al. (5), Khan et al. (59), and Latif et al. (15), presenting similar supporting results with the current study as 80% of the genotypes were G1 and G3. However, the current study shows the appearance of *E. equinus* (G4) and *E. ortleppi* (G5) for the first time from Pakistan, which might be due to the lack of complete molecular genotyping of *Echinococcus* in Pakistan; hence, it is not possible to evaluate the intensity of *E. ortleppi* and *E. equinus* infection in Pakistan. Reporting G4 and G5 infection in cattle increases the need for *Echinococcus* cyst genotyping to determine the infection pathology and pattern and to explore the most prevalent strain infecting humans and animals in the Pakistani population.

Animals were anatomically observed and visually inspected for the presence of hydatid cysts. During the visual inspection and organ palpation, only large cysts are visible to the naked eyes; however, very small cysts inside the organs (especially in the initial infection) are very difficult to find and report, which thus may lead to false-negative rates of CE. Therefore, anatomical observation accompanied by serological tests would provide more accurate information of CE prevalence in livestock. Furthermore, another limitation of the study is the small number of PCR samples used for phylogenetic analyses. Analyzing a large data set will further deepen our understanding of CE genetic diversity in the study area.

## Conclusion

The current study shows the presence of echinococcosis in livestock population of the Malakand division of Khyber Pakhtunkhwa, Pakistan. It also reveals various characteristics like sex, age, organs, and animal species that are at more risk for CE. The molecular analysis shows the high prevalence of G1-G3 genotypes among the livestock population; however, to the best of our knowledge, this is the first study in Pakistan that revealed the geographical distribution of G4 and G5 genotypes among slaughtered buffalo which act as an intermediate host. More genotyping is necessary to evaluate the contribution of G4 and G5 genotypes to the prevalence of human echinococcosis.

## Data Availability Statement

The original contributions presented in the study are included in the article/supplementary material, further inquiries can be directed to the corresponding authors.

## Ethics Statement

The animal study was reviewed and approved by the Ethical Approval Committee of COMSATS University, Islamabad under Reference No. CUI-Reg/Notif. 2255/19/2661.

## Author Contributions

AAS and IA: study planning. JK and NB: manuscript preparation. JK and SalK: data collection. JK, NB, and SalK: lab work and data analysis. SJ, SR, SNK, SanK, and RA: manuscript critical evaluation and final drafting. All authors approved the final draft of the manuscript.

## Funding

This study was supported by HEC under the Project No. 8085/Baluchistan/NRPU/R&D/HEC/2017, entitled Genomic and Proteomic Based Antigenic Characterization of Locally Prevalent Echinococcal Isolates for the Identification of Immunodominant Epitopes, Molecular Diagnostics Development, and Vaccine Design.

## Conflict of Interest

The authors declare that the research was conducted in the absence of any commercial or financial relationships that could be construed as a potential conflict of interest.

## Publisher's Note

All claims expressed in this article are solely those of the authors and do not necessarily represent those of their affiliated organizations, or those of the publisher, the editors and the reviewers. Any product that may be evaluated in this article, or claim that may be made by its manufacturer, is not guaranteed or endorsed by the publisher.
